# Kinetics of postdiagnosis platelet count with overall survival of pancreatic cancer: a counting process approach

**DOI:** 10.1002/cam4.644

**Published:** 2016-02-10

**Authors:** Yuanyuan Xiao, Hua Xie, Zhihui Xie, Zhenyi Shao, Wen Chen, Guoyou Qin, Naiqing Zhao

**Affiliations:** ^1^ Department of Biostatistics, School of Public HealthFudan UniversityShanghaiChina; ^2^ Health Information Center Shanghai Municipal Commission of Health and Family PlanningShanghaiChina; ^3^ School of Public HealthKunming Medical UniversityKunmingYunnanChina; ^4^ Key Lab of Health Technology AssessmentMinistry of Health (Fudan University)ShanghaiChina

**Keywords:** Counting process approach, pancreatic cancer, platelet count, survival

## Abstract

The association between long‐term variation of postdiagnosis platelets and survival of pancreatic cancer (PC) has never been discussed by using dynamic survival analysis method. In this retrospective study, we analyzed 311 histologically confirmed PC patients identified from a mega population‐based electronic inpatients database from 2012 to 2013 in China. Counting process approach was applied to restructure the original survival data, the association between post‐diagnosis platelet count and overall survival (OS) of PC was evaluated by multiple failure‐time Cox proportional hazards model. After counting process adjustment, multiple failure‐time Cox proportional hazards model revealed that, regardless of the treatment modalities PC patients received, postdiagnosis thrombocytopenia was prominently associated with OS, compared with PC patients with normally ranged platelet count, the HRs ranged from 2.04 (95% CI: 1.14–3.67) to 10.82 (95% CI: 2.63–44.54), and this inverse association was robust based on further sensitivity analysis. On the contrary, the association between thrombocytosis and OS of PC tended to be inconclusive. Our findings suggested that postdiagnosis thrombocytopenia was associated with significantly compromised survival among PC patients from this large retrospective cohort. Underlying mechanisms behind this association should be further investigated.

## Introduction

The prognostic value of circulating platelets in malignant tumors attracts lasting study interest 1, 2, 3, 4. In pancreatic cancer (PC), the association between platelet count and survival has also been discussed albeit at a lesser extent, yet like in other common cancers, conflicting results have been presented: a greater proportion of studies supported that thrombocytosis, an abnormally elevated level of platelet count, was associated with unfavorable survival of PC 5, 6, 7, while a few other studies reported the opposite or null association 8, 9.

Nearly all currently available studies on the association between platelet count and PC survival analyzed platelet count measured at baseline, mostly defined as at diagnosis or prior to cancer treatment. As a blood indicator, platelet count keeps changing constantly. In this case, compared with measurements from certain transient moments, the long‐term variation in platelet count after PC diagnosis and its influence on survival undoubtedly bear more significance in clinical practice.

On the basis of widely used Cox proportional hazards model, several extensions have been proposed to evaluate the effect of time‐varying or time‐dependent variables 10. The most intuitive way is to construct a function over time for time‐varying independent, and integrate this time function into Cox model with other constant covariates. Although the logic behind this method is simple, to correctly define this “time function” will be extremely difficult, if not impossible 11. Among all other methods, the counting process approach emphasizes restructuring of original data, puts weaker assumption on survival model itself, and presents a simpler way to analyze time‐varying covariates. Nevertheless, we can barely find its use in cancer survival literatures.

In this study, we aimed to evaluate the association between time‐varying platelet count measured after cancer diagnosis and overall survival (OS) of PC through the application of counting process approach and the subsequent multiple failure‐time Cox proportional hazards model, besides, we also compared results of multiple failure‐time Cox model with common Cox model which analyzed the association between platelet count measured at diagnosis and OS of PC.

## Material and Methods

### Study design

We performed a retrospective review in a mega population‐based electronic inpatients database. This database was established in the year of 2011 and has kept updating on daily basis ever since, all county‐level and above hospitals within Shanghai Metropolitan area, China, which are also qualified for cancer diagnosis, are responsible for tracking and reporting relevant information of all admitted patients, such as specifics of diagnosis, results of tests and examinations, and treatment details. Totally we identified 676 histologically confirmed PC patients from this database between 1 January 2012 and 31 December 2013. PC patients without baseline platelet count test result, which defined as within 30 days after the date of diagnosis, were excluded (*N *=* *339). A lag‐time period of 30 days was applied for the effect of platelet count can be revealed, and PC patients who died within the first month after cancer diagnosis were further removed (*N *=* *26). In the end, we included 311 PC patients for analysis.

The outcome of interest was OS, and the dates of death for PC patients were determined through external matching with death registration system. The matching deadline was set as 31 January 2015. The whole study was approved by Institutional Research Ethics Board of Fudan University.

### Variables and definitions

With regard to aforementioned definition of lag‐time, postdiagnosis platelet count was defined as consecutive measurements between the date of diagnosis and 30 days before the date of death for each included patient. We used 100–300 × 10^9^/L as the reference level for normal platelet count, thus thrombocytosis and thrombocytopenia were defined as platelet counts higher than 300 × 10^9^/L and lower than 100 × 10^9^/L, respectively. Other factors that need to be controlled for during analysis, like age at diagnosis, sex, and the adoption of curative resection or chemotherapy, were also extracted from this inpatients database simultaneously. Chemotherapy was defined as the administration of one or more following medications: gemcitabine, nab‐paclitaxel, 5‐fluorouracil, irinotecan, and oxaliplatin.

### Statistical analysis

The counting process approach in Cox proportional hazards model was originally proposed by Anderson and Gill 12 to deal with recurrent event and time‐varying covariates. When analyzing time‐varying covariates, this approach splits every single original observation into a group of “subobservations” at time points when a specific covariate varied. It assumes that the value of this time‐varying covariate stays put between two consecutive time points. Thus, within the transformed database, for every “subobservation,” all covariates will be static, and multiple failure‐time Cox proportional hazards model under different further assumptions, such as fixed or changed baseline hazard, constant or variant effect along with survival time, can therefore be applied. The major difference between multiple failure‐time Cox proportional hazard model and common Cox proportional hazards model is that, the former further adjusts for correlation between “subobservations” stemmed from the same original observation to get a robust variance, by using “sandwich” estimator for instance 13.

In this study, except for fixed baseline hazard, we applied counting process approach based on the assumption of constant effect of postdiagnosis platelet count on OS of PC. We latter performed a comprehensive sensitivity analysis: at first, we adopted the assumption that the effect of postdiagnosis platelet count was changing along with the survival time, and calculated interval‐specific effect estimate accordingly; then, we discussed the influence of diverse reference levels for defining normal platelet count in study results.

All statistical analyses were executed by SAS (version 9.2, SAS Institute Inc., Cary, NC,). The significance level was placed as two‐tailed probability < 0.05.

## Results

Basic characteristics of 311 PC patients we analyzed are listed in Table 1. The mean of age at diagnosis for all patients was 65.61 years, males and females were equivalent in number. A total of 67 patients went through curative resection, among them, 19 received distal pancreatectomy. Around one‐fifth patients received curative operation, nearly a half patients went through either palliative or adjuvant chemotherapy, based on whether curative operation was received. Among resected PC patients, no use of neoadjuvant chemotherapy was reported. Over 70% patients had a normal platelet count at diagnosis. Log‐rank test revealed that, although the median of survival length in PC patients with thrombocytosis was significantly longer, platelet count at diagnosis in general was not associated with OS of PC. By the time of matching deadline, 251 PC patients had experienced death, accounted for 80.71%.

**Table 1:** Basic characteristics of 311 PC patients analyzed

Characteristics	Overall(*N* = 311)	Platelet count at diagnosis			*P*
		Normal(*N *=* *235)	Thrombocytosis(*N* = 53)	Thrombocytopenia(*N *=* *23)	
Age at diagnosis (mean, SD.)	65.61 (10.72)	65.47 (10.85)	65.89 (11.03)	66.39 (9.84)	0.91
Sex (male, %)	156 (50.16)	124 (52.77)	19 (35.85)	13 (56.52)	0.07
Curative operation (yes, %)	67 (21.54)	52 (22.13)	14 (26.42)	1 (4.35)	0.07
Palliative or adjuvant chemotherapy (yes, %)	151 (48.55)	104 (44.26)	32 (60.38)	15 (65.22)	0.03
Survival length (median, days)	249	232	396	220	0.10

After further adjustment, multivariate Cox proportional hazards model revealed that, compared with normal platelet count, thrombocytosis at diagnosis was associated with prominently decreased hazard of death (HR: 0.66; 95% CI: 0.46–0.95), whereas thrombocytopenia showed nonsignificant influence on OS (Table 2).

**Table 2:** Crude and adjusted HRs for OS of PC related to multiple factors

Covariates	Univariate Cox model		Multivariate Cox model	
	Crude HR (95% CI)	*P*	Adjusted HR (95% CI)	*P*
Age at diagnosis (+ 5 years)	1.17 (1.09–1.24)	<0.01	1.13 (1.06–1.21)	<0.01
Sex (Male)	0.88 (0.69–1.13)	0.31	0.97 (0.75–1.26)	0.84
Curative operation				
Distal pancreatectomy	0.21 (0.09–0.48)	<0.01	0.26 (0.11–0.59)	<0.01
Other resection modalities	0.44 (0.30–0.64)	<0.01	0.48 (0.33–0.71)	<0.01
Palliative or adjuvant chemotherapy (Yes)	0.92 (0.72–1.18)	0.51	1.08 (0.83–1.41)	0.57
Thrombocytosis at diagnosis	0.72 (0.51–1.02)	0.07	0.66 (0.46–0.95)	0.03
Thrombocytopenia at diagnosis	1.22 (0.77–1.94)	0.39	1.00 (0.63–1.60)	0.99

We categorized 311 PC patients into four subgroups based on their treatment modalities: curative operation only, curative operation plus adjuvant chemotherapy, palliative chemotherapy only, and no treatment. Totally 2723 “subobservations” were generated from the patients by using counting process approach. Under the assumption of constant effect, after adjusted for age at diagnosis and sex, we found that: postdiagnosis thrombocytopenia was prominently associated with compromised survival in all four subgroups, compared with PC patients with normally ranged platelet count, the HRs ranged from 2.04 (95% CI: 1.14–3.67) to 10.82 (95% CI: 2.63–44.54), whereas an increased postdiagnosis platelet count only showed significant influence in survival in PC patients who received palliative chemotherapy (HR: 2.00; 95% CI: 1.10–3.63) (Table 3).

**Table 3:** Multiple failure‐time Cox model fitting results, counting process approach, constant effect assumption

Treatment modality	Adjusted HR^a^	95% CI	*P*
Curative operation only			
Postdiagnosis thrombocytosis	3.05	0.89–10.50	0.08
Postdiagnosis thrombocytopenia	10.82	2.63–44.54	<0.01
Curative operation + adjuvant chemotherapy			
Postdiagnosis thrombocytosis	0.93	0.15–5.67	0.93
Postdiagnosis thrombocytopenia	5.35	2.13–13.43	<0.01
Palliative chemotherapy only			
Postdiagnosis thrombocytosis	2.00	1.10–3.63	0.02
Postdiagnosis thrombocytopenia	3.79	2.39–6.01	<0.01
No treatment			
Postdiagnosis thrombocytosis	1.31	0.70–2.44	0.39
Postdiagnosis thrombocytopenia	2.04	1.14–3.67	0.02

a Adjusted for age at diagnosis, sex.

**Figure 1:**
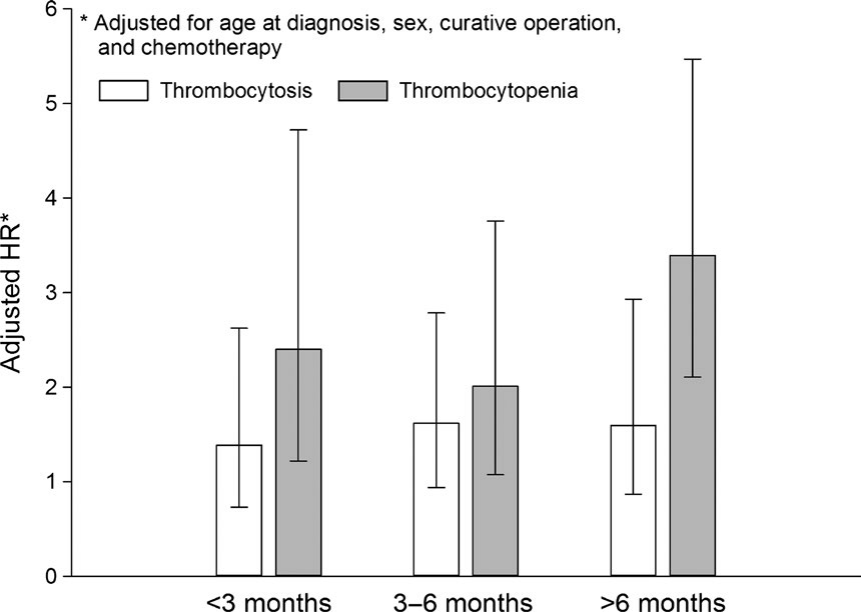
Adjusted HRs with 95% CIs for different platelet counts under variant effect assumption.

Survival time of PC patients was divided into the following three intervals: less than 3 months, 3–6 months, and above 6 months. Under the assumption of variant effect, counting process approach was applied separately within each interval. Multiple failure‐time Cox proportional hazards model indicated that, the associations between thrombocytosis and OS of PC in all three intervals were comparable but statistically insignificant, whereas the adjusted HR of thrombocytopenia stayed prominent, and dramatically increased in “above 6 months” survival interval (Fig. 1).

**Figure 2:**
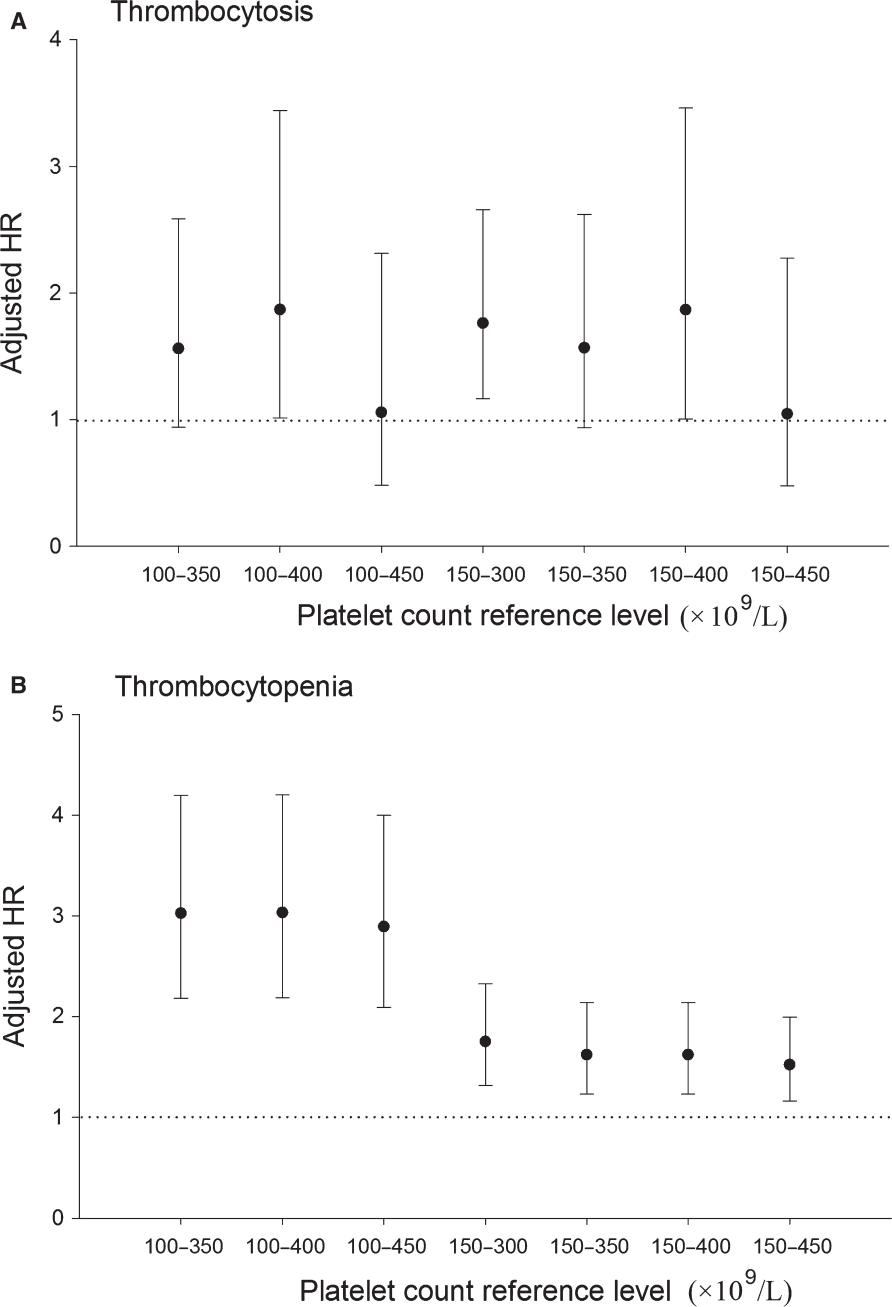
Adjusted HRs with 95% CIs by various platelet count reference levels. Both (A) and (B) were adjusted for age at diagnosis, sex, curative operation, and chemotherapy.

The cut‐offs up to 150 × 10^9^/L and 450 × 10^9^/L were also commonly used to define thrombocytopenia and thrombocytosis. Thus, we performed a sensitivity analysis based on different platelet count reference levels to check the robustness of study results. As we can learn from Figure 2, the choice of reference level did not influence statistical significance of the inverse association between thrombocytopenia and OS of PC, although the strength of association fluctuated.

## Discussion

In this retrospective cohort study, by applying counting process approach, we were able to discuss the influence of varying postdiagnosis platelet count on OS of PC. Based on analytical results, we found that regardless of treatment modalities, thrombocytopenia was consistently associated with significantly compromised survival of PC patients, and this inverse association was robust either with regard to different assumptions on the effect of platelet count, or to disparate reference levels of platelet count. On the contrary, the association between thrombocytosis and OS of PC tended to be inconsistent thus could not be concluded yet.

In PC patients, myelosuppressive chemotherapeutic regimens, especially gemcitabine, a predominantly administered first‐line medication for treatment of advanced PC, usually resulted in anemia, neutropenia, as well as thrombocytopenia 14, 15, 16. Other than this, because of anatomic vicinity, locally advanced PC can encroach upon portal vein or splenic vein, cause decreased liver function and hypersplenotrophy subsequently, both of which can lead to thrombocytopenia 14. Hence, it is possible that the association between thrombocytopenia and PC survival we found was confounded by those situations. However, a later performed subgroup analysis based on 259 PC patients revealed that, after further adjusted for simultaneously tested hemoglobin, neutrophil count, and albumin, a sensitive indicator of liver function, this association remained significant (Appendix 1). Although the possible influence of splenectomy on platelet count can be effectively controlled for, as we discriminated distal pancreatectomy from other resection modalities, for patients who did not perform splenectomy, the concurrent hypersplenism could not be addressed because of data limitation.

Abundant experimental evidences have been accumulated in supporting the detrimental effect of redundant platelets in cancer prognosis. For example, it has been verified that platelets can secret a series of proteins which promote tumor cell proliferation and angiogenesis directly 17, besides, platelets can also facilitate cancer metastasis through cloaking disseminated tumor cells and preventing them from being cleared by immune system 18. Therefore, although we did not find a stable association between thrombocytosis and PC survival, with regard to aforementioned major mechanisms, this issue warrants further investigation.

In our study, the prominent and stable inverse association between thrombocytopenia and PC survival probably suggested that, for pancreatic cancer, compared with thrombocytosis, it will be more urgent to cope with concurrent thrombocytopenia during the whole survival period in order to gain possible survival benefit. The anticancer propensity of platelets has been mentioned by a few in vitro studies. In early 2000s, Ahmad et al 19. discovered that upon proper activation, human platelets can induce apoptosis of tumor cells. More recently, Wang and Zhang 20 observed that murine platelets directly inhibited the growth of tumor cells. These findings illustrated that the role of platelets in cancer progression might not be as straightforward as previously anticipated. It is possible that the anticancer propensity of platelets was involved in the inverse association between thrombocytopenia and PC survival we found. Nevertheless, such hypothesis should be further validated.

As to the association between platelet count and PC survival, the results from multiple failure‐time Cox model and common Cox model were distinctively different. The latter disclosed that thrombocytosis at diagnosis was associated with significantly improved survival of PC, whereas thrombocytopenia showed nonsignificant influence. However, multiple failure‐time Cox proportional hazards model based on counting process approach on the contrary concluded a stable inverse association between postdiagnosis thrombocytopenia and OS of PC. Although intuitively, the results from two methods were not comparable, as the platelet counts they used were measured at different time points, the discrepancy itself requires close attention. It reminds us that in the field of cancer survival, when possible, dynamic survival methods should always be adopted to deal with time‐varying independents, marginal associations between measurements from certain transient moments and the outcome of interest could be misleading in guiding clinical practice.

Several limitations of this study should be considered. At first, when illustrating the influence of postdiagnosis platelet count on PC survival, compared with OS, indicators of PC progression are superior endpoints. However, because the indicators of PC progression were unavailable in this study, the association between sequential platelet count and PC progression could not be discussed. The possibility of residual confounding cannot be ruled out, especially, detailed information on tumor biology, current physical status and other possible causes of thrombocytopenia besides chemotherapy were unavailable. However, the acceptance of curative operation we included could be an ideal surrogate to tumor stage, meanwhile, by excluding patients who died within 30 days after PC diagnosis, the confounding of physical status can also be partly controlled for. Moreover, based on existing literature 21, the chance of other causes of thrombocytopenia among PC patients is comparatively low. Selection bias may introduce as PC patients with baseline platelet count only accounted for about a half of all histologically confirmed patients. Finally, because of data constraints, when performing multivariate analysis, other identified or possible prognostic factors of PC, such as tumor location, tumor size, pathological characteristics, postoperative complications, could not be adjusted, although we did not find solid evidences which suggest that those factors are also associated with platelet count variation in existing literature, once they were, residual confounding will inevitably be introduced.

In summary, our study found that a decreased postdiagnosis platelet count was consistently associated with significantly deteriorated OS of PC. This finding emphasizes the prognostic value of postdiagnosis platelets in PC survival. The underlying mechanisms behind this relationship should be explored for possible therapeutic intervention measures.

## Conflict of interest

None declared
